# Upper Temperature Limits of Tropical Marine Ectotherms: Global Warming Implications

**DOI:** 10.1371/journal.pone.0029340

**Published:** 2011-12-29

**Authors:** Khanh Dung T. Nguyen, Simon A. Morley, Chien-Houng Lai, Melody S. Clark, Koh Siang Tan, Amanda E. Bates, Lloyd S. Peck

**Affiliations:** 1 Tropical Marine Science Institute, National University of Singapore, Singapore, Singapore; 2 British Antarctic Survey, National Environmental Research Council, Cambridge, United Kingdom; 3 Institute of Marine and Antarctic Studies, University of Tasmania, Hobart, Tasmania, Australia; University of Sao Paulo, Brazil

## Abstract

Animal physiology, ecology and evolution are affected by temperature and it is expected that community structure will be strongly influenced by global warming. This is particularly relevant in the tropics, where organisms are already living close to their upper temperature limits and hence are highly vulnerable to rising temperature. Here we present data on upper temperature limits of 34 tropical marine ectotherm species from seven phyla living in intertidal and subtidal habitats. Short term thermal tolerances and vertical distributions were correlated, i.e., upper shore animals have higher thermal tolerance than lower shore and subtidal animals; however, animals, despite their respective tidal height, were susceptible to the same temperature in the long term. When temperatures were raised by 1°C hour^−1^, the upper lethal temperature range of intertidal ectotherms was 41–52°C, but this range was narrower and reduced to 37–41°C in subtidal animals. The rate of temperature change, however, affected intertidal and subtidal animals differently. In chronic heating experiments when temperature was raised weekly or monthly instead of every hour, upper temperature limits of subtidal species decreased from 40°C to 35.4°C, while the decrease was more than 10°C in high shore organisms. Hence in the long term, activity and survival of tropical marine organisms could be compromised just 2–3°C above present seawater temperatures. Differences between animals from environments that experience different levels of temperature variability suggest that the physiological mechanisms underlying thermal sensitivity may vary at different rates of warming.

## Introduction

Temperature is arguably one of the most important factors influencing the physiology, ecology and evolution of ectotherms [1], [2], with clear latitudinal and altitudinal influences on the distribution of species [3]. A sufficient and accurate understanding of how environmental change affects organisms requires detailed knowledge of how close species are to their thermal limits in nature, and how much spare capacity they possess to respond to further increases in habitat temperature [4], [5].

In terrestrial systems, extinction rates due to the loss of habitat are predicted to be severe and nonlinear, with losses increasing rapidly beyond a 2°C rise, compounded by other interactive physical and biological factors [3]. An increase of just 2–3°C was also found to be detrimental to tropical mangrove molluscs [6]. These limits may be reached very soon as the global average temperature has already risen *ca*. 0.74^°^C over the past century (1906–2005) [7] and is expected to increase between 1.4°C and 5.8°C over the remainder of this century [8]. This might lead to ecosystem level perturbations in the tropics where biodiversity is greatest, and also where ectotherms have one of the greatest risks of extinction, due to reduced tolerance to further warming, limited acclimation ability, and reduced dispersal and settlement [5], [9], [10], [11]. The pattern of declining thermal safety margins with decreasing latitude, from temperate to tropical regions, is common for a range of ectotherms, including insects, lizards, turtles, frogs [5], [10], [12] and marine porcelain crabs [4]. This mirrors the reduction in environmental variability from temperate to tropical regions [13] and is explained by the reduced physiological flexibility of organisms that have evolved in more thermally stable environments [14], [15]. These hypotheses also apply to Antarctic marine ectotherms which are thermal specialists living in a stable, permanently cold environment where temperature elevation of only 2°C above current maximum seawater temperature is predicted to be detrimental to many species [16]. Furthermore the most sensitive Antarctic marine species to warming, the brittle star *Ophionotus victoriae* cannot withstand a 2°C experimental temperature rise [17]. Hence, tropical and Antarctic marine ectotherms live in very stable thermal environments and they are expected to suffer significant reduction in their fitness with rising seawater temperature [5], [9], [16]. However, the intertidal is a highly variable environment with a gradient of temperature stress from the high shore to the subtidal, but also local factors, such as the timing of summer spring low waters, may override any latitudinal signal [18], [19]. It is therefore important to measure experienced micro-habitat temperatures in different habitats to compare with organism thermal tolerance.

Understanding the physiological mechanisms underlying organism sensitivity to temperature, and whether this varies with the rate of warming and between environments, will help improve predictions of organism vulnerability to environmental variation. Oxygen has long been understood to play a role in setting the acute thermal limits of aquatic organisms [9]. The concept of oxygen- and capacity-limited thermal tolerance provides a framework for a consistent physiological mechanism underlying species thermal limit that ultimately determines the temperature sensitivity of ecological relationships and fitness [20], [21]. However, a study of the effects of the rate of temperature change on the thermal limits of a range of Antarctic marine ectotherms suggested that different mechanisms limit thermal tolerance at different rates of temperature change [16]. This was supported by a meta-analysis of the thermal tolerance of temperate marine ectotherms where seasonal curves were not parallel but diverged more at slower rates of thermal challenge [22] which is in contrast to the expectation of oxygen and capacity limited thermal tolerance [20].

In this study, we examined 34 tropical marine invertebrate species belonging to seven phyla from upper and lower intertidal shores as well as subtidal habitats in Singapore, to establish the sensitivities of their upper temperature limit (UTL) relative to current microhabitat temperature. Using different rates of temperature change has proved to be a powerful technique to extrapolate the results of laboratory experiments, which have generally been conducted at fast rates of change, into more ecologically realistic time scales [16]. Thermal limits have previously been correlated with body size and activity (after [16]) to specifically test if aerobic scope and the principles of oxygen and capacity limitation (*cf* [23]) were linked to thermal limits. These correlations will be investigated for tropical ectotherms in this study.

## Materials and Methods

* Ethic statement: N/A (The collection and experimentation on the invertebrates used in this study does not require a permit)

A total of 34 marine invertebrate species in seven phyla (Mollusca, Crustacea, Polychaeta, Sipuncula, Brachiopoda, Echinodermata and Ascidiacea) were used in this study. Organisms were collected from intertidal shores on St. John's Island (1.22°N, 103.85°E), mudflats at Kranji Reservoir Park (1.26°N, 103.75°E) and subtidal habitats in the vicinity of Changi (1.40°N, 103.98°E) in Singapore. The only brachiopod in our experiments, *Lingula anatina*, was collected from the low intertidal mudflat in Phuket, Thailand (7.99°N, 98.33°E). Tropical marine intertidal species can have a wide vertical distribution on the shore so specimens were collected from a restricted habitat where adults were most abundant. Intertidal animals were divided into upper intertidal (UIT) and lower intertidal (LIT) groups. Subtidal (SubT) species were dredged or trapped from 5 to 15 m depth.

Temperatures experienced by animals in their natural microhabitats were recorded for each habitat using two temperature loggers for a period of at least three months. In all experiments, animals were collected and held in a flow-through aquarium system at ambient temperature (29.4^°^C±0.2^°^C) and 12∶12 h light: dark lighting regime for 24–48 h prior to being used. Specimens damaged during collection or appearing unhealthy were not used in experiments.

Collected animals were divided into two groups:the treatments were subjected to temperature control at different rates of change and the controls were kept in the flow-through aquarium system at 29.4 ^°^C (±0.2^°^C) until the end of each experiment. Both treatment and control animals were fed twice a week. Temperature control methods and regimes were based on Peck *et al.* [16]. Four rates of warming were used in experiments: 1^°^C hour^−1^ (±0.1^°^C, n = 34 where n is the number of species tested in each treatment, n_i_ = 20 where n_i_ is the number of individuals per species tested), 1^°^C day^−1^ (±0.3°C, n = 33, n_i_ = 20), 2.5^°^C week^−1^ (±0.3^°^C, n = 19, n_i_ = 20) and 3^°^C month^−1^ (±0.5^°^C, n = 5, n_i_ = 20). Temperatures were raised incrementally with regular monitoring of mortality at each 1°C step. In the 3^°^C month^−1^ experiment, separate groups of animals were maintained for a period of 90 days or until more than 50% mortality had occurred at two elevated temperatures of 32.4°C and 35.4^°^C. The starting temperature for all experiments was 29.4°C, which was the mean seawater temperature measured at 1 m depth around Singapore [24]. Experiment tanks were vigorously aerated, and animals were always kept underwater. Mortality of the controls (<10%) only occurred in three species during 2.5^°^C week^−1^ experiments (*Siphoneria guamensis, Babylonia areolata* and *Lasaea* sp.) and only one species during 1^°^C day^−1^ experiment (*Volachlamys singaporina*).

Different tactile or behavioural stimuli were employed to determine the upper limit of each species (Table S1). When the animals were no longer responsive to external stimuli, the temperature was noted and individual size was measured with vernier calipers to the nearest ±0.1 mm. For most species, the maximum linear dimension was measured. For echinoderms, the length of the longest arm of the starfish *Archaster typicus* and the brittle star *Ophiactis savignyi*, and the test diameter of the sea urchin *Temnopleurus toreumaticus*, were measured. The effect of size on UTL was analysed through correlation analysis within each species (following Peck *et al.* [16]). Using the ranking system of Peck *et al.* [16], the activity quotient of each species was calculated, based on four major activity components: feeding mode, type, speed and duration of movement each day (Table 1).

**Table 1:** Scores for feeding mode, movement type, speed and duration during day for each species.

Habitat	Species	Feeding mode	Movement type	Movement speed	Movement duration	Product	Activity quotient
**UIT**	*Echinolittorina malaccana*	3	3	2	2	36	2.45
	*Planaxis sulcatus*	3	3	2	2	36	2.45
	*Nerita lineata*	3	3	3	4	108	3.22
	*Siphonaria guamensis*	3	3	2	2	36	2.45
	*Amphibalanus amphitrite*	2	2	3	5	60	2.78
	*Patelloida saccharinoides*	3	3	2	2	36	2.45
	*Xenostrobus atratus*	2	2	2	3	24	2.21
	*Mytilopsis sallei*	2	2	2	2	16	2
	*Cerithidea cingulata*	3	3	3	3	81	3
	*Batillaria zonalis*	3	3	3	3	81	3
	*Atactodea glabrata*	2	4	3	2	48	2.63
	*Dotilla myctiroides*	4	4	4	5	320	4.23
**LIT**	*Archaster typicus*	3	3	2	2	36	2.45
	*Phascolosoma arcuatum*	3	4	2	3	72	2.91
	*Onchidium tumidum*	3	3	2	3	54	2.71
	*Gari elongata*	2	4	2	2	32	2.38
	*Diopatra neapolitana*	2	4	3	3	72	2.91
	*Laternula truncata*	2	4	2	2	32	2.38
	*Laternula boschasina*	2	4	2	2	32	2.38
	*Lingula anatina*	1	4	2	2	16	2
	*Isognomon ephippium*	2	4	2	2	32	2.38
	*Lasea sp.*	2	4	2	2	32	2.38
**SubT**	*Myomenippe hardwickii*	4	5	4	5	400	4.47
	*Perna viridis*	2	2	3	4	48	2.63
	*Euchelus tricingulatus*	3	3	2	3	54	2.71
	*Barbatia trapezina*	2	2	2	2	16	2
	*Ophiactis savignyi*	3	3	3	3	81	3
	*Corbula crassa*	2	2	2	2	16	2
	*Temnopleurus toreumaticus*	3	3	3	2	54	2.71
	*Pyura sp.*	2	1	1	1	2	1.19
	*Volachlamys radula*	2	2	3	2	24	2.21
	*Thais echinata*	3	3	2	3	54	2.71
	*Morula funicular*	3	3	2	3	54	2.71
	*Babylonia areolata*	3	3	2	5	90	3.08

The activity quotient is derived as the fourth root of the product of the feeding and activity scores (based on [16]).

In feeding mode:1 = passive ciliary, 2 = pumping, 3 = grazing, 4 = capture.

In movement type:1 = sedentary, 2 = sedentary + muscular activity, 3 = crawling, 4 = burrowing, 5 = walking, 6 = swimming.

In movement speed:1 = none, 2 = slow, 3 = medium, 4 = fast.

In movement duration:1 = never, 2 = very rare, 3 = occasional, 4 = sometimes, 5 = often.

The relationships between upper temperature tolerance, experimental rate of temperature increase (-log transformed to meet the assumption of linearity), habitat and activity quotient [16] were tested by including fixed and random effects using a linear modelling approach. Initially a full set of explanatory variables were included as fixed effects based on a priori hypotheses. Nested taxonomy was included as a random effect on the model intercept.

Selection of the optimal model was in two steps. Firstly including taxonomy as a random effect was justified using a top-down strategy [25]. A full model including first and second order terms (fixed component) was fitted using restricted maximum likelihood estimation (REML) and models compared without (generalized linear least squares, gls) and with a random effect (linear mixed effects models, lme). Taxonomy was included as a nested random effect as follows: Species in Genus in Family in Order in Class in Phylum. Each taxonomic level was removed sequentially and compared using the Akaike's information criterion (AIC). The model with the lowest AIC value was retained. An F-test was used to confirm that the random effect explained a significant portion of the variance (Table S3). Second, to identify the minimum adequate model, non-significant fixed factors were removed following a step-wise procedure [25]. The minimum adequate model was then identified using AIC and F-tests for models fitted using maximum likelihood (ML).

Linear model assumptions were met by checking normalized residual plots for homogeneity of variance. All analyses were conducted using the nLME package in R (v. 2.13.1).

## Results

**Figure 1:**
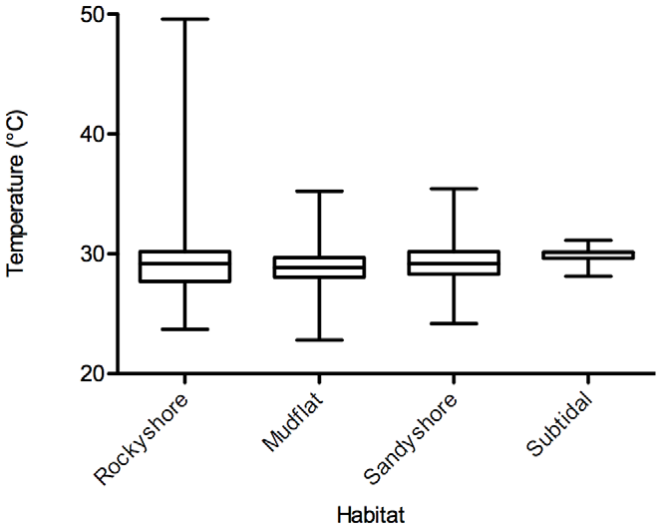
Temperature profiles of major habitats in Singapore. The box-whisker plots show maximum, minimum, mean and 95 percentile temperatures from temperature logger data deployed at each habitat for at least three months.

The mean temperature of the Singapore shoreline is close to 30°C (Figure 1). However, microhabitat temperature differed greatly depending on the substratum and tidal height. Temperature loggers deployed on the upper rocky shore surface recorded the greatest magnitude and variation in temperature exceeding 50°C on hot days. Temperatures recorded over a three month period from our collection sites at sandy shores and mudflats ranged from 25° to 35°C, but exceptionally reached 40°C for short periods in some days. However, there was no significant difference in the mean and range of habitat temperatures experienced by infaunal organisms in sandy and muddy substrata, and hence they were both classified as lower intertidal habitat in our study. Subtidal habitats had a more stable range of between 28 and 31°C. The average daily mean and daily max temperatures of the three habitats were significantly different (ANOVA, Mean temperature: F_3,356_ = 41.37, *p*<0.01; Max temperature: F_3,356_ = 131.8, *p*<0.01). There was a significant difference in the temperature variability between different environments (Bartlett's test, *p*<0.01).

### Upper thermal limits in acute and chronic heating experiments

**Figure 2:**
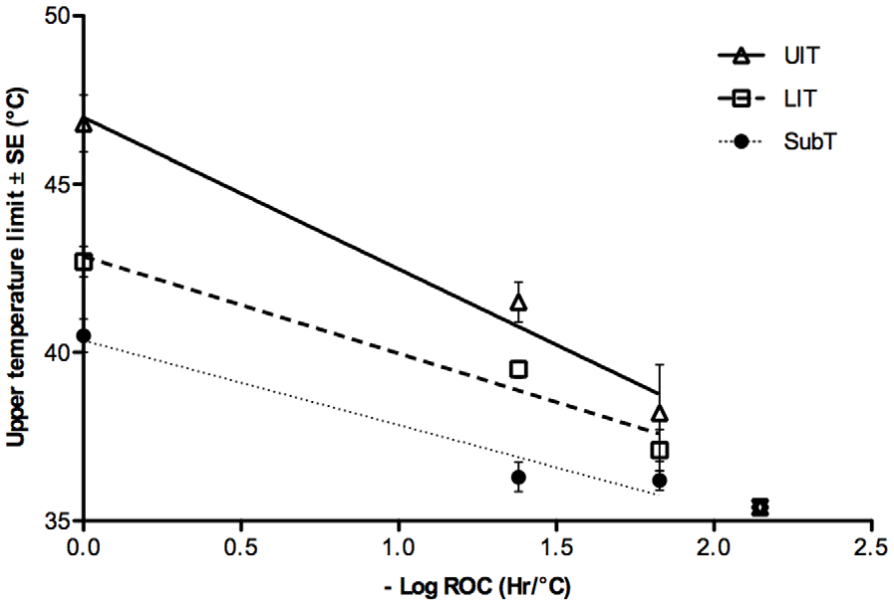
Effect of rate of temperature change on ULTs (Mean±SE) of animals from different habitats: UIT (open triangle), LIT (open square) and SubT (filled circle). Error estimates for parameters are in Tables 2 and S3). UIT: y = −4.22x+46.95 LIT: y = −2.80x+42.87 SubT: y = −2.67x+40.41.

For each rate of temperature increase, the mean of the average UTLs of every species from the same habitat were calculated and presented as one data point in Figure 2, to give a value representative of all species from that habitat. The average UTL of each species was listed in Table S1. The UTLs of the animals in this study varied with their vertical position on the shore according to tidal height (Figure 2, Table 2). For 1^°^C hour^−1^ experiments (-log scale  = 0), UTLs were 6.54 °C higher in the upper intertidal versus subtidal (t = 8.40, *p*<0.01) and 2.45 °C higher in the lower intertidal versus subtidal (t = 3.15, *p*<0.01). However, despite being significantly different, the thermal limits of both UIT, LIT and SubT organisms were about 10°C above their current mean maximum habitat temperature (UIT:37–38°C, LIT:33°C, SubT:30.5°C).

**Table 2:** Summary of minimum adequate linear mixed effects (lme) model results for upper lethal temperature (ULT) as a function of log_10_(experimental rate of temperature change) and habitat (subtidal, low intertidal and upper intertidal).

Fixed-effects	df	Contrast coefficient	Standard error	t-value	P-value	Random-effects	% variance
reference	49	40.41	0.54	75.13	<0.0001	S (77)	62.40
**rate**	**49**	−**2.66**	**0.27**	−**9.94**	**<0.0001**	Residual	37.60
habitat(LIT)	31	2.45	0.78	3.15	0.0036		
habitat(UIT)	31	6.54	0.78	8.40	<0.0001		
**rate: habitat(LIT)**	**49**	−**0.13**	**0.39**	−**0.33**	**0.75**		
**rate: habitat(UIT)**	**49**	−**1.57**	**0.41**	−**3.85**	**0.0003**		

Treatment contrasts indicate the effect of each parameter level on the reference level (subtidal). Species (S) was retained as a random effect on the intercept. Effect types are intercept (unshaded) and slope (shaded). The model-averaged coefficient estimates and 95% confidence intervals for all parameters included in the full model are in Table S3.

lme(UTL∼rate*habitat,random = S)

AICc  = 335.14

LIT  =  lower intertidal, UIT = upper intertidal, AICc  =  *Akaike's information criterion* corrected for finite sample sizes, df  =  degrees of freedom.

The thermal tolerance of the 34 species tested was strongly reduced at slower rates of warming (rate contrast coefficient: −2.66, t = −9.94, *p*<0.01; Figure 2, Table 2), and this was particularly apparent for UIT animals. The slope of relationship between UTL and rate of warming was significantly lower for the high shore treatment (rate:habitat(UIT) contrast coefficient: −1.57, t = −3.85, p <0.01). Thus, at slower rates of increase the least difference in UTL between high intertidal and subtidal invertebrates was detected. By contrast, the difference in UTL between lower intertidal and subtidal animals was consistent for different rates of warming (rate:habitat(LIT) contrast coefficient: −0.13, t = −0.33, *p* = 0.75).

Few species could withstand temperatures above 40°C in chronic heating trials when the warming rate was slower than 2.5°C week^−1^. The UTLs of upper intertidal species decreased by more than 10°C from the 1^°^C hour^−1^ values when thermal stress was prolonged for weeks while there was only a 5°C decline for subtidal species. 3^°^C month^−1^ experiments were only performed on a single lower intertidal species and four subtidal species (n_i_ = 20). After three months, all the controls (n_i_ = 20) were still healthy. At 35.4°C, the sea urchin *Temnopleurus toreumaticus* died within four weeks, followed by the green mussel *Perna viridis,* and two subtidal bivalves *Barbatia trapezina* and *Corbula crassa* which died in weeks 4 and 5, respectively. The low intertidal starfish *Archaster typicus* survived longer than the subtidal species, until week 6. At 32.4°C, all animals were alive and appeared healthy except for *T. toreumaticus.* The spines of this sea urchin started to drop off after one month, indicating that this species could not fully acclimate and was in a time limited physiological condition at this temperature. This suggested its long-term survival limit was lower, between 29.4 and 32.4°C.

Once the random effect of species was accounted for, there was no significant effect of activity quotient on UTLs. Moreover, the interactions between the rate of temperature change and shore height with activity quotient were not retained in the optimal model (Table 2). The unconditional 95% confidence limits for these parameters also contained zero (see Table S3).

### Upper thermal limits and body size

Our test species ranged from the very small bivalve *Lasaea* sp., 0.5–3.5 mm in size, to larger species such as the green mussel *Perna viridis* and the starfish *Archaster typicus*, both which exceeded 100 mm in shell length and arm length, respectively. Most species in our study were less than 50 mm in diameter or length with six species smaller than 10 mm. The coefficients of variation in size within species were generally less than 20% and rarely more than 30% (Table 3). There was no consistent relationship between UTL and body size observed from our data. The linear regression slopes were either negative (17 and 12 species in 1^°^C hour^−1^ and 1^°^C day^−1^ respectively), i.e., smaller individuals were able to tolerate higher temperature than the larger ones of the same species, or positive (10 spp. for both 1^°^C hour^−1^ and 1^°^C day^−1^), i.e., larger animals had higher UTLs; or zero (seven 1^°^C hour^−1^ spp. and five 1^°^C day^−1^ spp.), i.e., all samples died at the same temperature. Statistical analysis of our dataset did not yield significant regressions, except for four species in 1^°^C hour^−1^ (three negative and one positive) and two species in 1^°^C day^−1^ (one negative and one positive) (Table 3).

**Table 3:** Regression parameters for equations relating VST and ST mean UTLs for quartiles calculated on size ranges (mm) of studied species.

	Species	Size (mm)	CV (%)	VST				ST			
				Slope	r^2^	P	F	Slope	r^2^	P	F
**UIT**	*E. malaccana*	5–8	14	−0.16	0.14	0.62	0.33	0.06	0.05	0.77	0.11
	*P. sulcatus*	8–40	20	0	-	-	-	-0.03	0.15	0.62	0.34
	*N. lineata*	10–22	26	−0.09	0.29	0.46	0.82	−0.02	0.08	0.72	0.17
	*S. guamensis*	4–10	23	−0.13	0.24	0.52	0.61	−0.26	0.69	0.17	4.53
	*A. amphitrite*	3–5	15	0.02	0.005	0.93	0.01	0.75	0.78	0.12	6.90
	*P. saccharinoides*	12–18	10	−0.05	0.09	0.69	0.21	0	-	-	-
	*X. atratus*	4–9	22	−0.32	0.94	0.03	31.44	0	-	-	-
	*M. sallei*	16–22	11	0	-	-	-	0.07	0.09	0.71	0.19
	*C. cingulata*	12–25	14	−0.09	0.96	0.02	43.33	-	-	-	-
	*B. zonalis*	11–28	19	−0.04	0.93	0.03	27.24	-	-	-	-
	*A. glabrata*	14–35	22	−0.01	0.07	0.73	0.15	−0.06	0.96	0.02	46.20
	*D. myctiroides*	8–25	9	0	-	-	-	-	-	-	-
**LIT**	*A. typicus*	45–90	16	0.004	0.02	0.85	0.05	0.01	0.27	0.48	0.72
	*P. arcuatum*	75–135	19	−0.01	0.59	0.23	2.93	−0.002	0.05	0.77	0.11
	*O. tumidum*	9–17	20	0.01	0.01	0.91	0.02	-	-	-	-
	*G. elongata*	24–41	25	−0.05	0.64	0.20	3.58	−0.005	0.02	0.86	0.04
	*D. neapolitana*	28–54	16	−0.03	0.51	0.28	2.09	-	-	-	-
	*L. truncata*	21–42	18	0.01	0.001	0.97	0.002	0.01	0.08	0.72	0.17
	*L. boschasina*	9–20	19	0.06	0.05	0.78	0.10	−0.21	0.48	0.31	1.82
	*L. anatina*	31–48	9	0.25	0.99	0.001	749.9	0.04	0.03	0.82	0.07
	*I. ephippium*	29–52	19	−0.0007	0.003	0.95	0.005	−0.01	0.86	0.07	11.95
	*Lasaea sp.*	0.5–3.5	41	−0.04	0.15	0.61	0.34	0	-	-	-
**SubT**	*M. hardwickii*	13–55	42	0.02	0.36	0.59	0.56	-	-	-	-
	*P. viridis*	77–104	9	0	-	-	-	0.01	0.53	0.27	2.27
	*E. tricingulatus*	6–12	18	0	-	-	-	0	-	-	-
	*B. trapezina*	20–26	7	0.04	0.05	0.78	0.10	−0.1	0.08	0.71	0.18
	*O. savignyi*	6–22	39	0.01	0.05	0.78	0.10	0.19	0.97	0.02	64.43
	*C. crassa*	10–22	17	0.15	0.83	0.09	10.04	−0.10	0.39	0.38	1.25
	*T. toreumaticus*	21–37	12	−0.02	0.26	0.49	0.69	−0.01	0.14	0.62	0.33
	*Pyura sp.*	10–40	10	0	-	-	-	-	-	-	-
	*V. radula*	26–37	15	−0.004	0.0004	0.98	0.001	−0.01	0.25	0.50	0.65
	*T. echinata*	22–36	12	−0.0004	0.0002	0.99	0.0002	0	-	-	-
	*M. funicula*	18–30	13	0	-	-	-	0.003	0.01	0.95	0.007
	*B. areolata*	32–49	7	−0.002	0.001	0.96	0.003	0.15	0.79	0.11	7.45

Mean UTLs were calculated and regressed against mean size for each size quartile. Co-efficient of variation (CV) in size was also computed for each species.

## Discussion

### Upper thermal limits and vertical distribution

The heat tolerance of marine intertidal animals is related to their vertical distribution along the shore [6], [26], [27], [28]. This correlation was clear in our results where UTLs decreased from upper to lower intertidal, and were lowest in subtidal animals. Our data also showed clear separation between the upper lethal temperature ranges of the three habitats, though there were some outliers with uncharacteristically high or low UTLs for their habitat.

The most extreme example of an elevated UTL was *Echinolittorina malaccana*, a littorinid gastropod found on the upper intertidal regions of most rocky shores across the Indo-Pacific [29]. The 1^°^C hour^−1^ and 1^°^C day^−1^ limits of this species were 3 to 4°C higher than other intertidal animals used in this study, including those found in the same tidal zone. It has been shown that *E. malaccana* can regulate its metabolism at high temperatures and enter a state of protective metabolic depression at temperatures above 30°C [30]; as well as maintain enzyme (glutamate oxaloacetate transaminase) activity at very high temperature (55°C) [31]. For species living in the upper eulittoral fringe, elevated thermal tolerance is employed together with other passive mechanisms such as foot withdrawal, shell nodulation, hinging behaviour, position maintenance, aestivation in air and metabolic depression to raise their UTLs [30], [32], [33]. These unique adaptations might be responsible for their unusually high UTL.

At the opposite end, the upper intertidal crab *Dotilla myctiroides* had a UTL which fell within the range of the subtidal group (Table S2). This discrepancy can be explained by the burrowing and “igloo”-constructing behaviour of this soldier crab [34], which allows the crab to continually dig deeper into the sand until it is below the water level assuring water uptake and temperature regulation [35]. Therefore, its experienced microhabitat temperatures are more representative of a subtidal existence and its experimental temperature range is thus closer to subtidal species than other intertidal species.

### Upper thermal limits and rate of temperature change

Our data clearly showed that the UTLs of upper intertidal species decreased more rapidly as rates of heating were slowed compared to subtidal species. Although having much higher 1^°^C hour^−1^ temperature limits, intertidal animals actually live as close to their thermal limits as subtidal species, i.e. both had the same 1^°^C hour^−1^ thermal safety margin of 10°C above their average maximal habitat temperature (Figures 1 and 2). A similar thermal safety margin was reported previously for a few tropical marine invertebrates [9]. However, in 2.5°C week^−1^ experiments, upper intertidal species only had a thermal safety margin of about 3°C, whereas in low intertidal species, the value was 4°C and in wholly subtidal species this was 6°C.

There is a developing pattern, broadly supported by this study, that animals which live in rapidly changing thermal environments such as the upper shore in the tropics have steeper relationships between thermal limits and the rate of temperature change than those living in less variable environments in the lower or subtidal shore. Different mechanisms may be employed and prioritized at different rates of change [36]. Adaptations that improve survival in the short term might not be sufficient or may even reduce the fitness of organisms facing long term thermal stress. Hence, intertidal organisms that are adapted to cope with highly variable environments and therefore require high acute heat tolerances may actually be more sensitive to chronic warming rates compared to subtidal species. High intertidal species may even be more vulnerable if their ability to adapt, via changes in the genetic structure of populations is relatively limited [37] or if behavioural or ecological factors militate against them [36]. Moreover, the outliers in this study also emphasized the importance of taking microhabitat conditions and adaptive strategies into account when assessing thermal tolerances, especially for animals living in extreme conditions, or active species with complex behaviours (non-climatic adaptation [38], [39], [40]). A recent meta-analysis of literature reporting the effect of different rates of temperate change on the lethal limits of temperate species also found that the slope of the relationship changed across environments and after seasonal acclimatization [22]. Along with the current results for tropical ectotherms there is a strong suggestion that different mechanisms vary in importance at different rates of change (e.g. Peck *et al.* [16]) and care must be taken when interpreting the evolutionary significance of findings from thermal assays (e.g. Rezende *et al.* [41]).

### Upper thermal limits, individual size and activity

Several studies have shown that within a species, smaller animals have a higher temperature tolerance than larger conspecifics, as predicted by the principle of oxygen and capacity limitation [16], [21], [26], [42], [43]. In our study however, there was little or no consistency in the relationship between body size and UTL. This may have been because the size range used in this study was not large enough for an underlying relationship to be apparent. Other studies, however, have also failed to find a correlation between body size and thermal tolerance, especially at slow heating rate [44] including an increase in thermal tolerance with size, which was found in the temperate beachflea *Orchestra gammarellus* [45]. Recent comparisons of the thermal tolerance of different sized individuals of the tropical bivalves *L. boschasina* and *L. truncata* were equivocal (Morley *et al.,* unpublished data). Juvenile *L. boschasina* had a significantly higher 1^°^C hour^−1^ thermal limit than adults (42.6±0.6 versus 40.9±1.4; t = 4.7, p<0.01) but juvenile *L. truncata* had the same thermal limits as adults (42.5±0.7 versus 42.0; t = 0.9, p = 0.4). A relationship between size and UTL may therefore not be universal and require further and more careful investigation, especially in the tropics where animals tend to be smaller compared to those at higher latitudes [46].

Activity level can be used as a proxy for aerobic scope and can therefore test the principle that species with higher aerobic scope will have a greater physiological capacity to cope with elevated temperatures, leading to a higher lethal limit [16], [20]. However activity did not vary across rates of experiment warming, with heat tolerance, or with shore height (Table S3). Thus, higher aerobic scope may not generally lead to enhanced physiological capacity in tropical species, or, if present, this relationship may have been obscured by the taxonomic or habitat variability in the data set. A future research direction will be to assess if higher aerobic scope relates to greater heat tolerance in a related group of marine invertebrates from tropical latitudes.

### Conclusion

Our study provides the first data of the UTLs of tropical marine animals across different rates of temperature change that can be compared with that published data for temperate and polar species. Our data reinforce the suggestion that 1) animals living in thermally stable environments have reduced acclimatory ability, and 2) animals living constantly close to their upper limits in aseasonal environments are particularly susceptible to increases in temperature. This vulnerability, combined with the fact that regions nearer to the equator and the poles have faster warming rates compared to the global average [47], [48], [49] which can be more than 1°C in 50 years [50], animals in both environments are possibly the most vulnerable and likely to be the first affected under current global warming and climate change conditions. Range shifts are already being recorded in a wide variety of marine [51], [52], [53] and terrestrial [54], [55], [56] species and understanding the mechanisms underlying these changes is of critial importance to enable us to predict how ecosystems will change into the future.

## Supporting Information

Table S1 Mean UTLs of 34 species from different habitats under four heating regimes VST, ST, MT and LT (see methods): 11 UIT spp., 11 LIT spp. and 12 SubT spp. The types of tactile and/or behavioural stimuli performed on each species to determine their response are listed below with (1)- Body movement and muscle contraction, (2)- Siphon reaction, (3)- Ability to hold the shell closed, (4)- Tube-feet or arms/spines movement, (5)- Response of the mouth and cirri, (6)- Response of legs and mouthparts.
(DOCX)

Table S2 Model comparisons with and without inclusion of species. Inclusion of taxonomic signal significantly improved the model, as indicated by a likelihood ratio greater than one. AIC  =  *Akaike's information criterion*; df  =  degrees of freedom.
(DOCX)

Table S3 Multimodel inference produced model-averaged parameter estimates and unconditional errors based on AICc for all variables included in the full linear mixed effects (lme) model: upper lethal temperature (ULT) as a function of three fixed factors: log(experimental rate of temperature change), habitat (subtidal, littoral, upper intertidal), and activity quotient. First and second order terms were included in the full model based on *a priori* hypotheses. Treatment coefficients contrast each variable level with the reference level (subtidal). Effect types are intercept (unshaded) and slope (shaded). Starred parameters indicate contrast coefficients with 95% confidence intervals greater than 0. The minimum adequate model results and % variance explained by the random effect of “Species” are in Table 1.
(DOCX)
