# Supplementary material for: Upper Temperature Limits of Tropical Marine Ectotherms: Global Warming Implications
Source: PLoS One. 2011 Dec 29;6(12):e29340. doi: 10.1371/journal.pone.0029340 (PMC3248430; doi:10.1371/journal.pone.0029340)
Supplement: Table S1 — Mean UTLs of 34 species from different habitats under four heating regimes VST, ST, MT and LT (see methods): 11 UIT spp., 11 LIT spp. and 12 SubT spp. The types of tactile and/or behavioural stimuli performed on each species to determine their response are listed below with (1)- Body movement and muscle contraction, (2)- Siphon reaction, (3)- Ability to hold the shell closed, (4)- Tube-feet or arms/spines movement, (5)- Response of the mouth and cirri, (6)- Response of legs and mouthparts. (DOCX) [file pone.0029340.s001.docx]

**SUPPORTING INFORMATION**

**Table S1**. Mean UTLs of 34 species from different habitats under four heating regimes VST, ST, MT and LT (see methods): 11 UIT spp., 11 LIT spp. and 12 SubT spp. The types of tactile and/or behavioural stimuli performed on each species to determine their response are listed below with (1)- Body movement and muscle contraction, (2)- Siphon reaction, (3)- Ability to hold the shell closed, (4)- Tube-feet or arms/spines movement, (5)- Response of the mouth and cirri, (6)- Response of legs and mouthparts

| **Group** | | **Habi** | **Species** | **Sti-** | **UTL (^˚^C)** | | | |
| --- | --- | --- | --- | --- | --- | --- | --- | --- |
|  |  | **-tat** |  | **muli** | **VST** | **ST** | **MT** | **LT** |
| **Molusca** | **Gastro-poda** | UIT | *Echinolittorina malaccana* Philippi, 1847 (Littorinidae) | 1 | 51.4 | 43.6 | 42.0 | - |
|  |  |  | *Planaxis sulcatus* Born, 1791 (Planaxidae) | 1 | 49.4 | 43.6 | - | - |
|  |  |  | *Nerita lineata* Gmelin, 1791 (Neritidae) | 1 | 48.8 | 42.1 | 38.9 | - |
|  |  |  | *Siphonaria guamensis* Quoy & Gaimard, 1833 (Siphonariidae) | 1 | 45.4 | 39.6 | 36.5 | - |
|  |  |  | *Patelloida saccharinoides* Habe & Kosuge, 1966 (Lottiidae) | 1 | 44.9 | 39.4 | 35.6 | - |
|  |  |  | *Cerithidea cingulata* Gmelin, 1791 (Potamididae) | 1 | 48.9 | 43.5 | - | - |
|  |  |  | *Batillaria zonalis* Bruguière, 1792 (Batillariidae) | 1 | 48.2 | 43.4 | - | - |
|  |  | LIT | *Onchidium tumidum* Semper, 1885 (Onchidiidae) | 1 | 44.8 | - | - | - |
|  |  | SubT | *Euchelus tricingulatus* A. Adams, 1851 (Chilodontidae) | 1 | 41.4 | 36.4 | 36.9 | - |
|  |  |  | *Thais echinata* Blainville, 1832 (Muricidae) | 1 | 41.5 | 36.6 |  |  |
|  |  |  | *Morula funicula* Wood, 1828 (Muricidae) | 1 | 42.4 | 36.5 | 36.5 |  |
|  |  |  | *Babylonia areolata* Link, 1807 (Babyloniidae) | 1 | 41.1 | 34.8 | 36.7 |  |
|  | **Bivalvia** | UIT | *Xenostrobus atratus* Lischke, 1871 (Mytilidae) | 3 | 43.5 | 39.4 | - | - |
|  |  |  | *Atactodea glabrata* Gmelin, 1791 (Mesodesmatidae) | 3 | 47.4 | 42.9 | - | - |
|  |  | LIT | *Mytilopsis sallei* Récluz, 1849 (Dreissenidae) | 3 | 42.4 | 39.7 | 38.4 | - |
|  |  |  | *Gari elongata* Lamarck, 1818 (Psammobiidae) | 3 | 43.0 | 39.3 | 36.0 | - |
|  |  |  | *Laternula truncata* Lamarck, 1818 (Laternulidae) | 3 | 42.0 | 39.4 | - | - |
|  |  |  | *Laternula boschasina* Reeve, 1863 (Laternulidae) | 3 | 40.9 | 38.8 | - | - |
|  |  |  | *Isognomon ephippium* Linnaeus, 1758 (Isognomonidae) | 3 | 44.3 | 40.5 | 37.0 | - |
|  |  |  | *Lasaea* sp. (Lasaeidae)*.* | 3 | 42.5 | 39.4 | 34.4 |  |
|  |  | SubT | *Perna viridis* Linnaeus, 1758 (Mytilidae) | 3 | 41.4 | 37.6 | 36.3 | 35.4 |
|  |  |  | *Barbatia trapezina* Lamarck, 1819 (Arcidae) | 3 | 40.4 | 36.7 | 35.2 | 35.4 |
|  |  |  | *Corbula crassa* Reeve, 1843 (Corbulidae) | 3 | 40.1 | 36.4 | 37.3 | 35.4 |
|  |  |  | *Volachlamys singaporina* Sowerby, 1842 (Pectinidae) | 3 | 37.1 | 33.7 | - | - |
| **Echinodermata** | | LIT | *Archaster typicus* Roschel, 1840 (Archasteridae) | 4 | 41.5 | 39.8 | 37.2 | 35.4 |
|  |  | SubT | *Ophiactis savignyi* Müller & Troschel, 1842 (Ophiactidae) | 4 | 40.2 | 35.9 | 34.9 | - |
|  |  |  | *Temnopleurus toreumaticus* Leske, 1778 (Temnopleuridae) | 4 | 39.3 | 36.5 | 36.0 | 35.4 |
| **Crustacea** | | UIT | *Amphibalanus amphitrite* Darwin, 1854 (Balanidae) | 5 | 45.0 | 40.9 | - | - |
|  |  |  | *Dotilla myctiroides* H. Milne Edwards, 1852 (Dotillidae) | 6 | 42.4 | 38.8 | - | - |
|  |  | SubT | *Myomenippe hardwickii* Gray, 1831  (Menippidae) | 6 | 43.2 | 39.7 | - | - |
| **Sipuncula** | | LIT | *Phascolosoma arcuatum* Gray, 1828 (Phascolosomatidae) | 1 | 45.4 | 41.2 | 37.0 | - |
| **Polychaeta** | | LIT | *Diopatra neapolitana* Delle Chiaje, 1841 (Onuphidae) | 1 | 42.4 | 38.8 | - | - |
| **Ascidiacea** | | SubT | *Pyura* sp*.* (Pyuridae) | 2 | 38.1 | 34.9 | - | - |
| **Brachiopoda** | | LIT | *Lingula anatina* Lamarck, 1801 (Lingulidae) | 3 | 40.7 | 38.7 | 39.5 | - |
