# Supplementary material for: Upper Temperature Limits of Tropical Marine Ectotherms: Global Warming Implications
Source: PLoS One. 2011 Dec 29;6(12):e29340. doi: 10.1371/journal.pone.0029340 (PMC3248430; doi:10.1371/journal.pone.0029340)
Supplement: Table S2 — Model comparisons with and without inclusion of species. Inclusion of taxonomic signal significantly improved the model, as indicated by a likelihood ratio greater than one. AIC = Akaike's information criterion; df = degrees of freedom. (DOCX) [file pone.0029340.s002.docx]

**Table S2.** Model comparisons with and without inclusion of species. Inclusion of taxonomic signal significantly improved the model, as indicated by a likelihood ratio greater than one. AIC = *Akaike’s information criterion*; df = degrees of freedom.

| **Fixed-effects** | **df** | **AIC** | **log likelihood** | **likelihood ratio** | **P-value** |
| --- | --- | --- | --- | --- | --- |
| without taxonomy^1^  with taxonomy^2^ | 11  12 | 350.78  330.74 | -164.39  -153.37 | 22.04 | <0.0001 |

^1^lme(UTL~(rate*habitat)+(rate*activity)+(habitat*activity),random=S)

^2^gls(UTL~(rate*habitat)+(rate*activity)+(habitat*activity))
