# Supplementary material for: Upper Temperature Limits of Tropical Marine Ectotherms: Global Warming Implications
Source: PLoS One. 2011 Dec 29;6(12):e29340. doi: 10.1371/journal.pone.0029340 (PMC3248430; doi:10.1371/journal.pone.0029340)
Supplement: Table S3 — Multimodel inference produced model-averaged parameter estimates and unconditional errors based on AICc for all variables included in the full linear mixed effects (lme) model: upper lethal temperature (ULT) as a function of three fixed factors: log(experimental rate of temperature change), habitat (subtidal, littoral, upper intertidal), and activity quotient. First and second order terms were included in the full model based on a priori hypotheses. Treatment coefficients contrast each variable level with the reference level (subtidal). Effect types are intercept (unshaded) and slope (shaded). Starred parameters indicate contrast coefficients with 95% confidence intervals greater than 0. The minimum adequate model results and % variance explained by the random effect of “Species” are in Table 1. (DOCX) [file pone.0029340.s003.docx]

**Table S3.** Multimodel inference produced model-averaged parameter estimates and unconditional errors based on AICc for all variables included in the full linear mixed effects (lme) model: upper lethal temperature (ULT) as a function of three fixed factors: log(experimental rate of temperature change), habitat (subtidal, littoral, upper intertidal), and activity quotient. First and second order terms were included in the full model based on *a priori* hypotheses. Treatment coefficients contrast each variable level with the reference level (subtidal). Effect types are intercept (unshaded) and slope (shaded). Starred parameters indicate contrast coefficients with 95% confidence intervals greater than 0. The minimum adequate model results and % variance explained by the random effect of “Species” are in Table 1.

**lme(UTL~(rate*habitat)+(rate*activity)+(habitat*activity),random=S)**

| **Fixed-effects** | **Contrast coefficient** | **Standard error** | **Lower 95% interval** | **Upper 95% interval** |
| --- | --- | --- | --- | --- |
| **rate*** | **-2.57** | **0.52** | **-1.54** | **-3.59** |
| habitat(LIT) | 2.68 | 2.24 | -1.72 | 7.09 |
| habitat(UIT)* | 8.04 | 3.07 | 2.01 | 14.07 |
| activity | 1.02 | 0.63 | -0.22 | 2.26 |
| **rate: habitat(LIT)** | **-0.13** | **0.38** | **0.61** | **-0.88** |
| **rate: habitat(UIT)*** | **-1.56** | **0.39** | **-0.79** | **-2.33** |
| **rate:activity** | **-0.14** | **0.32** | **0.50** | **-0.77** |
| habitat(LIT):activity | -0.19 | 1.64 | -3.41 | 3.04 |
| habitat(UIT):activity | -2.13 | 1.08 | -4.25 | -0.01 |

LIT = lower intertidal, UIT=upper intertidal
